# Predicting the mechanical hip–knee–ankle angle accurately from standard knee radiographs: a cross-validation experiment in 100 patients

**DOI:** 10.1080/17453674.2020.1779516

**Published:** 2020-06-22

**Authors:** Willem Paul Gielis, Hassan Rayegan, Vahid Arbabi, Seyed Y Ahmadi Brooghani, Claudia Lindner, Tim F Cootes, Pim A de Jong, H Weinans, Roel J H Custers

**Affiliations:** a Department of Orthopedic Surgery, UMC Utrecht, Utrecht, The Netherlands; b Department of Mechanical Engineering, Faculty of Engineering, University of Birjand, Birjand, Iran; c Department of Biomechanical Engineering, Faculty of Mechanical, Maritime, and Materials Engineering, Delft University of Technology (TU Delft), Delft, The Netherlands; d Division of Informatics, Imaging & Data Sciences, University of Manchester, Manchester, UK; e Department of Radiology, UMC Utrecht and Utrecht University, Utrecht, The Netherlands

## Abstract

Background and purpose — Being able to predict the hip–knee–ankle angle (HKAA) from standard knee radiographs allows studies on malalignment in cohorts lacking full-limb radiography. We aimed to develop an automated image analysis pipeline to measure the femoro-tibial angle (FTA) from standard knee radiographs and test various FTA definitions to predict the HKAA.

Patients and methods — We included 110 pairs of standard knee and full-limb radiographs. Automatic search algorithms found anatomic landmarks on standard knee radiographs. Based on these landmarks, the FTA was automatically calculated according to 9 different definitions (6 described in the literature and 3 newly developed). Pearson and intra-class correlation coefficient [ICC]) were determined between the FTA and HKAA as measured on full-limb radiographs. Subsequently, the top 4 FTA definitions were used to predict the HKAA in a 5-fold cross-validation setting.

Results — Across all pairs of images, the Pearson correlations between FTA and HKAA ranged between 0.83 and 0.90. The ICC values from 0.83 to 0.90. In the cross-validation experiments to predict the HKAA, these values decreased only minimally. The mean absolute error for the best method to predict the HKAA from standard knee radiographs was 1.8° (SD 1.3).

Interpretation — We showed that the HKAA can be automatically predicted from standard knee radiographs with fair accuracy and high correlation compared with the true HKAA. Therefore, this method enables research of the relationship between malalignment and knee pathology in large (epidemiological) studies lacking full-limb radiography.

The mechanical axis of the lower limb, which determines knee (mal)alignment, is historically measured using the hip–knee–ankle-angle (HKAA), an angle between the mechanical axes of the femur and tibia. The femoral axis runs through the centers of the femoral head and knee joint. The tibial axis runs through the centers of the knee and ankle joints (Figure 1).

**Figure 1.**
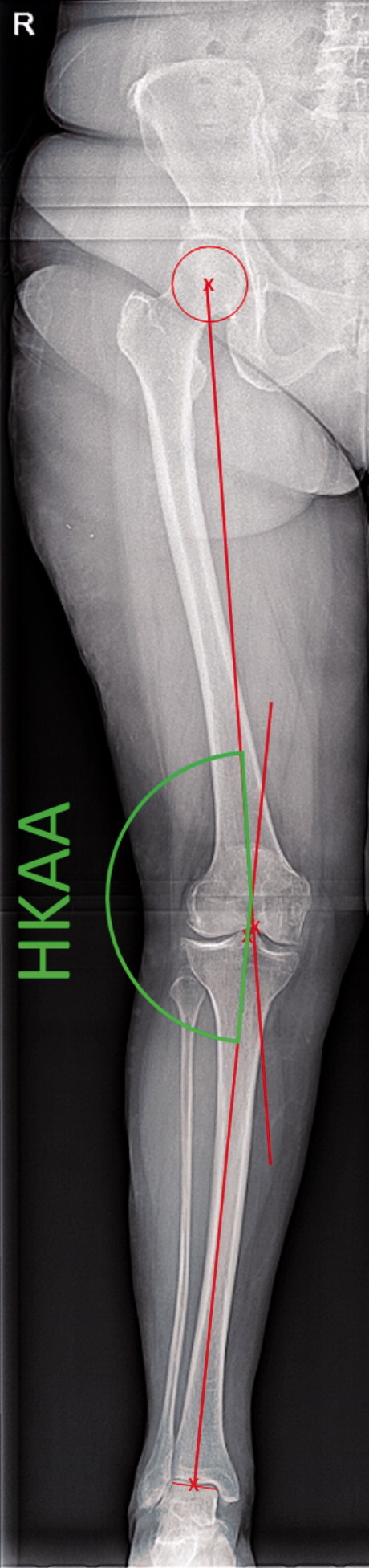
Measurement of the Hip–knee–ankle angle on full limb radiograph. The hip–knee–ankle angle (HKAA, in green) is measured between 2 axes (in red). One axis runs from the middle of the femoral head to the middle of the femoral notch, and a second axis from the middle of the tibial notch to the middle of the talar head. Definitions of the femoral axis from left to right (top): Fem1—mid-shaft at approximately 10 cm proximal of the femoral notch + mid-shaft in the area where the meta- and epiphysis meet (van Raaij et al. 2009, Iranpour-Boroujeni et al. 2014, Zampogna et al. 2015). Fem2—mid-shaft at approximately 10 cm proximal of the femoral notch + center of the femoral notch (Felson et al. 2009, van Raaij et al. 2009, McDaniel et al. 2010, Sheehy et al. 2011). Fem3—mid-shaft at approximately 10 cm proximal of the femoral notch + base of the tibial spines (Kraus et al. 2005, Hinman et al. 2006, Issa et al. 2007, McDaniel et al. 2010, Navali et al. 2012, Iranpour-Boroujeni et al. 2014, Zampogna et al. 2015). Fem4—mid-shaft at approximately 10 cm proximal of the femoral notch + middle of tibial plateau (McDaniel et al. 2010). Definitions of the tibial axis from left to right (bottom): Tib1—mid-shaft at approximately 10 cm distal of the base of the tibial spines + mid-shaft in the area where the meta- and epiphysis meet (van Raaij et al. 2009, Iranpour-Boroujeni et al. 2014, Zampogna et al. 2015). Tib2 Mid-shaft at approximately 10 cm distal of the base of the tibial spines + center of the femoral notch (McDaniel et al. 2010). Tib3—mid-shaft at approximately 10 cm distal of the base of the tibial spines + base of the tibial spines (Kraus et al. 2005, Hinman et al. 2006, Issa et al. 2007, Colebatch et al. 2009, van Raaij et al. 2009, McDaniel et al. 2010, Sheehy et al. 2011, Navali et al. 2012, Iranpour-Boroujeni et al. 2014, Zampogna et al. 2015). Tib4—mid-shaft at approximately 10 cm distal of the base of the tibial spines + middle of tibial plateau (McDaniel et al. 2010). The 2 pictures on the left show the measurement of the FTA using method 2 for the femoral axis and method 1 for the tibial axis on a standard AP knee radiograph from the present data set.

A standard knee radiograph is one of the primary tools in the diagnostic process of knee complaints and it is undertaken for a majority of patients. Correspondingly, many epidemiological studies focusing on the knee include standard knee radiographs. However, to verify and measure involvement of malalignment in the pathophysiology, the HKAA should be measured. This requires a full-limb radiograph (Figure 1). Compared with a standard knee radiograph, full-limb radiography involves higher costs, the need for specialized equipment, and a larger effective radiation dose for the patient. These are important reasons for knee OA cohort studies not to include full-limb radiographs. As standard knee radiographs are available for the majority of patients with knee complaints and participants of epidemiological knee-focused studies, it is desirable to have a method for defining knee (mal)alignment from a standard knee radiograph.

The femoro-tibial angle (FTA), an angle between the anatomic axes of the femur and tibia (Figure 2), can be used to predict the mechanical axis from a standard knee radiograph. The FTA is an important measurement that can predict the development of knee OA (Brouwer et al. 2007, Moyer et al. 2016). Multiple definitions for the FTA have been proposed (Table 1) (Kraus et al. 2005, Hinman et al. 2006, Brouwer et al. 2007, Issa et al. 2007, Colebatch et al. 2009, Felson et al. 2009, van Raaij et al. 2009, McDaniel et al. 2010, Sheehy et al. 2011, Navali et al. 2012, Zampogna et al. 2015). However, a direct comparison between all FTA definitions on the same data is lacking. Additionally, no studies used cross- or external validation to confirm results. As such, there is no consensus on which FTA definition should be used to predict the HKAA.

**Figure 2.**
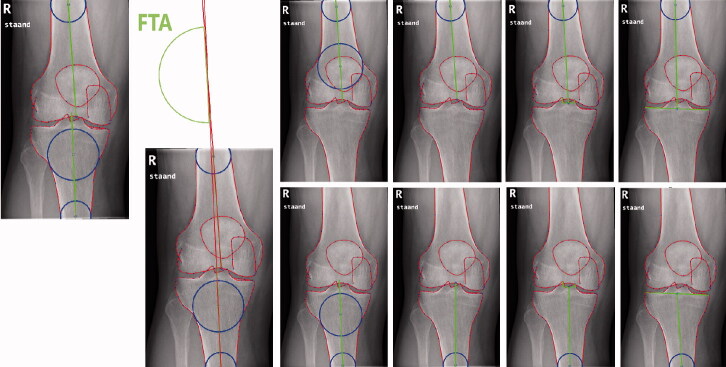
Measurement of the femoro-tibial angle (FTA) on standard knee radiographs. Tib4 Fem4 Tib3 Fem3 Tib2 Fem2 Tib1 Fem1

**Table 1.** Correlations between femoro-tibial angle and hip–knee–ankle angle as reported in the literature

Landmarks	Radiograph	Pearson correlation	Reference
Fem3 + Tib3	AP extended	0.26	Zampogna et al. 2015
Fem1 + Tib1	AP extended	0.71	
Fem3 + Tib3	AP extended	0.81	Colebatch et al. 2009
Fem2 + Tib2	PA semi-flexed **^a^**	0.50	McDaniel et al. 2010
Fem3 + Tib3	PA semi-flexed **^a^**	0.65	
Fem4 + Tib4	PA semi-flexed **^a^**	0.55	
Fem3 + Tib3 **^b^**	PA semi-flexed **^a^**	0.64	
Fem2 + Tib3	PA semi-flexed **^a^**	0.59	
Fem3 + Tib3	PA semi-flexed **^a^**	0.86	Issa et al. 2007
Fem2 + Tib3	PA semi-flexed **^a^**	0.66	Felson et al. 2009
Fem1 + Tib1 **^c^**	PA semi-flexed **^a^**	0.76	Iranpour-Boroujeni et al. 2014
Fem3 + Tib3	PA semi-flexed **^a^**	0.68	
Fem3 + Tib3	PA semi-flexed **^a^**	0.75	Kraus et al. 2005
Fem3 + Tib3	Full-limb	0.65	
Fem3 + Tib3	Full-limb	0.88	Hinman et al. 2006
Fem2 + Tib3	Full-limb	0.34	van Raaij et al. 2009
Fem1 + Tib1	Full-limb	0.65	
Fem2 + Tib3	Full-limb	0.88	Sheehy et al. 2012
Fem3 + Tib3	Full-limb	0.93	Navali et al. 2012

**^a^**Positioning aided with Synaflexer frame.

**^b^**Slight variation where the tips of the tibial spines are used instead of the base.

**^c^**Slight variation where the bottom point at the femur is determined using the middle femoral condyles instead of the shaft.

In addition to morphological measurements, statistical shape modelling is used in OA research to quantify variation in joint shape. Multiple studies showed the shape of a joint is a major factor in the incidence and progression of OA (Haverkamp et al. 2011, Waarsing et al. 2011, Agricola et al. 2015). A key step in the statistical shape modelling process is to outline the structures of interest in the medical images (e.g., radiographs) using anatomical landmarks. Manually placed landmarks on a set of radiographs can be used to train automated search models to place the respective points on new unseen images automatically, paving the road to analyzing large datasets (Lindner et al. 2013a, 2013b). Furthermore, the landmark positions obtained by the search models can easily be used to calculate morphologic measurement such as joint space width or the FTA.

The ability to predict the HKAA using automated FTA measurements from standard knee radiographs would make studies on malalignment feasible in large cohorts that lack full-limb radiography. This study aimed (i) to develop an automated image analysis pipeline to measure the FTA from a standard knee radiograph; and (ii) to analyze the performance of various FTA definitions in predicting the HKAA as measured on a full-limb radiograph. 

## Patients and methods

### Patients

We included 100 full-limb (50 males) radiographs, acquired for clinical care at the department of Orthopaedic Surgery of the UMC Utrecht, the Netherlands, in a consecutive series between March and November 2017. All patients were 40 years or older. For inclusion at least 1 standard knee radiograph made on the same day had to be available. We excluded patients with femoral and tibial deformities due to fractures, surgeries (including joint replacement of knee, hip, or ankle, and osteotomies) and developmental disorders. When radiographs of both legs were available for 1 subject, both were included in the study.

### Radiographic acquisition

Weight-bearing extended anteroposterior full-limb radiographs, with the patella facing straight towards the X-ray tube, were undertaken. On the same day, weight-bearing extended anteroposterior knee radiographs with the patella facing forward were taken. Standard knee radiographs were assessed by WPG for Kellgren–Lawrence (KL) grades. Before the assessment, the rater completed the tutorial for KL grading by Hayes et al. (2016). The tutorial includes 19 cases and an answer sheet to test the effect of the tutorial. The square weighted kappa for inter-rater reliability between WPG and the answer sheet was 0.969.

### Alignment measurements

The mechanical HKAA was used as gold standard and was measured on the full-limb radiographs (Moreland et al. 1987). An axis was drawn from the middle of the femoral head to the center of the femoral notch. A second axis was drawn from the base of the tibial spines to the center of the ankle joint. The HKAA was defined as the angle between these axes (Figure 1) (Sharma et al. 2001).

The FTA was measured on standard knee radiographs as the angle between the axis of the femur and tibia. We used a bespoke search model in BoneFinder (www.bone-finder.com, Centre for Imaging Sciences, University of Manchester, UK) to automatically outline the distal femur, patella, and proximal tibia using 111 landmarks (Lindner et al. 2013b). All automatically obtained landmarks were checked and manually corrected if necessary. The identified landmarks were used to automatically calculate the FTA. For measuring the femoral and tibial axes, 4 definitions each were considered based on previous literature (Figure 2) (Kraus et al. 2005, Hinman et al. 2006, Brouwer et al. 2007, Issa et al. 2007, Colebatch et al. 2009, Felson et al. 2009, van Raaij et al. 2009, McDaniel et al. 2010, Sheehy et al. 2011, Navali et al. 2012, Zampogna et al. 2015). 9 combinations between the femoral and tibial axes measurements were used to calculate the FTA (Fem1+Tib1, Fem1 + Tib3, Fem1 + Tib4, Fem2 + Tib1, Fem2 + Tib2, Fem2 + Tib3, Fem2 + Tib4, Fem3 + Tib3, Fem4 + Tib4). A varus angle is displayed as a negative number, a valgus angle as a positive number. As the standard knee radiographs were not calibrated for absolute distance, we used the width of the femoral condyles and tibial plateau to place circles needed for FTA measurements at approximately 10 cm from the joint line (Figure 2). Based on data from previous work we used a ratio of 1.52 for the femur and 1.42 for the tibia (Wesseling et al. 2016). We used the center of a circle touching the medial and lateral cortex to determine the mid-shaft points.

### Statistics

We used Pearson correlation coefficients to study which FTA method has the strongest correlation with the HKAA. Across all image pairs, we predicted the HKAA from the FTA using linear regression models. A simple model using only 1 FTA predictor, a model including a quadratic term, and a model including sex were considered. Using these predictions, we calculated 2-way mixed single-measures intra-class correlation coefficients for absolute agreement (ICC) between predicted HKAA and observed HKAA.

For the 4 FTA definitions with the strongest correlation to the HKAA, we performed 5-fold cross-validation experiments on the same data set. We randomly distributed the dataset in 5 parts, each accounting for 20% of the cases. In each fold, we calculated a linear regression formula to predict the HKAA based on the FTA using 80% of the data and used it to predict the HKAA in the remaining 20% of cases. We repeated this process 5 times, so each case will have a predicted HKAA. For this dataset, we present the Pearson correlation and ICC between the predicted HKAA and gold standard. In addition, we present a Bland–Altman plot displaying absolute measurement errors of predicted HKAA vs. the gold standard in our cross-validation experiments. As no similar experiment was published previously, a valid sample-size calculation was not possible and we applied the minimum of 100 cases as suggested by Vergouwe et al. (2005). We chose to include 50 males and 50 females, to account for sex-specific differences.

### Ethics, funding, and potential conflicts of interest

All radiographs were anonymized, and a waiver of consent was obtained from the local medical ethical committee (no. 17-760/C). This work was supported by Reuma Nederland (LLP-22) and the APPROACH project. APPROACH has received support from the Innovative Medicines Initiative Joint Undertaking under Grant Agreement no. 115770, resources of which are composed of a financial contribution from the European Union’s Seventh Framework Programme (FP7/2007-2013) and an EFPIA companies’ in-kind contribution. See www.imi.europa.eu. C. Lindner and T.F. Cootes were funded by the Engineering and Physical Sciences Research Council, UK (EP/M012611/1) and by the Medical Research Council, UK (MR/S00405X/1). Drs Cootes and Lindner have a patent US 9928443, EP 2893491 issued.

## Results

Of 100 full-limb radiographs, 11 had radiographs of both knees available and 89 had only 1 knee radiograph available. 1 knee radiograph was of insufficient quality to perform FTA measurements and was excluded, resulting in 110 full-limb/standard knee radiograph pairs. The mean age was 54 (SD 7.4) and 53 knees were male. Of all knees, 9 were KL 0, 23 were KL 1, 30 were KL 2, 36 were KL3, and 12 were KL 4.

### Correlation between FTA and HKAA measurements across all pairs of images

Across all pairs of images, the Pearson correlations between FTA and HKAA ranged between 0.83 and 0.90 (Table 2). The ICC values ranged from 0.83 to 0.90. The best correlations between HKAA and FTA measurements were found using the FTA defined as a femoral axis between the mid-shaft of the femur (approximately 10 cm above the joint line) and the femoral notch (Fem2), and a tibial axis running through 2 points in the mid-shaft of the tibia (approximately 4 cm and 10 cm beneath the tibial plateau (Tib1). Linear regression to predict the HKAA using the optimal FTA method (Fem2 + Tib1) produced the formula: HKAA = –2.182 + FTA*0.995. The mean absolute error between the predicted HKAA and the observed HKAA was 1.7° (SD 1.2°, range 0.1–5.4).

**Table 2.** Pearson correlation coefficients and intra-class correlations (ICC) between FTA and HKAA measurements (across all pairs of images)

Method	Pearson correlation	ICC (95% CI)
Fem1 + Tib1	0.88	0.87 (0.82–0.91)
Fem1 + Tib3	0.86	0.86 (0.80–0.90)
Fem1 + Tib4	0.86	0.86 (0.80–0.90)
Fem2 + Tib1	0.90	0.90 (0.85–0.93)
Fem2 + Tib2	0.87	0.86 (0.80–0.90)
Fem2 + Tib3	0.89	0.89 (0.84–0.92)
Fem2 + Tib4	0.89	0.89 (0.84–0.92)
Fem3 + Tib3	0.84	0.83 (0.76–0.88)
Fem4 + Tib4	0.83	0.82 (0.74–0.87)

### Correlation between FTA and HKAA predictions in cross-validation experiments

**Figure 3.**
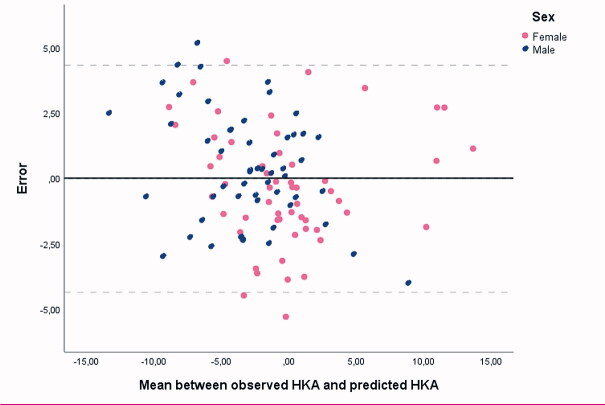
Bland–Altman plot depicting the error between the observed HKAA (gold standard) and the predicted HKAA in the cross-validation setting. Negative numbers represent the degree of varus alignment and positive numbers represent the degree of valgus alignment. The solid line depicts the mean error and the dotted lines the 95% confidence interval.

The correlation statistics found in the cross-validation setting were comparable to those found across all pairs of images, albeit minimally weakened (Table 3). Again, the combination of femoral axis 2 and tibial axis 1 showed the best correlation (Pearson correlation 0.90, ICC 0.90). The mean absolute error between the predicted HKAA and the observed HKAA was 1.8° (SD 1.3°, range 0.1–5.3) in the cross-validation setting. The Bland–Altman plot depicts the error between the observed HKAA (gold standard) and the predicted HKAA in the cross-validation setting (Figure 3). No systematic errors or outliers were found in this plot. Although females were more likely to have a valgus alignment compared with males, the error between observed HKAA and predicted HKAA was similar between sexes (p = 0.9). Linear regression models containing an interaction between FTA and sex, or a quadratic term, performed slightly better across all pairs of images, but performed slightly worse in the cross-validation experiments (data not shown).

**Table 3.** Pearson correlation coefficients and intra-class correlations (ICC) between FTA and HKAA measurements (cross-validation experiments)

Method	Pearson correlation	ICC (95% CI)
Fem1 + Tib1	0.88	0.87 (0.82–0.91)
Fem2 + Tib1	0.90	0.90 (0.85–0.93)
Fem2 + Tib3	0.89	0.89 (0.84–0.92)
Fem2 + Tib4	0.89	0.87 (0.80–0.91)

## Discussion

This study showed that the mechanical HKAA can be predicted from a standard knee radiograph using our automated pipeline. We used several FTA definitions and compared their performance in predicting the HKAA. The best-performing FTA definition used a femoral axis between the mid-shaft of the femur (approximately 10 cm above the joint line) and the femoral notch, and a tibial axis running through 2 points in the mid-shaft of the tibia (approximately 4 cm and 10 cm beneath the tibial plateau). This combination to measure FTA had not been reported in the literature.

Compared with previous work, the Pearson correlation coefficient between FTA and HKAA measurements (0.83 to 0.90) was high across all pairs of images (Tables 1 and 2) (Kraus et al. 2005, Hinman et al. 2006, Brouwer et al. 2007, Issa et al. 2007, Colebatch et al. 2009, Felson et al. 2009, van Raaij et al. 2009, Sheehy et al. 2011, Navali et al. 2012, Zampogna et al. 2015). The results of the cross-validation can be used to estimate the performance of the HKAA predictions in new cases. In the cross-validation the Pearson correlation between the automatically calculated FTA and the predicted HKAA was 0.90. The mean absolute error was 1.8° (SD 1.3°). To the best of our knowledge the performance of the predicted HKAA based on FTA has not been reported in any cross- or external validation studies.

The automatically calculated FTA provides an easy tool to study the influence of varus/valgus malalignment in OA cohorts or trials for which standard knee radiographs are available. We tested only the FTA produced by our automatic analysis pipeline to predict mechanical HKAA. However, the pipeline may be used to collect other measurements automatically and enables the rapid analysis of a collection of measurements for a large number of radiographs. The search algorithms we used were trained on only a small database (293 knees). Small corrections to the landmarks had to be made, costing approximately 1 minute per radiograph. In the future we expect the search model to have sufficient accuracy to run fully automatically without the need for manual correction. A database containing around 1,000 knees should be sufficient to achieve this (Gielis et al. 2020).

While the standard AP radiograph is most often used in the clinics, numerous OA studies use a semi-flexed PA radiograph. This technique aims to compensate for the tibial slope and give a more accurate reading on the joint space width. The generalizability of our methods to PA radiographs should be checked, and the formula to calculate the predicted HKAA may need adaptation. Additionally, some of the FTA definition may be applied in knees with a prosthesis, notably the definition using only landmarks in the femoral and tibial shafts. However, due to prosthesis placement, translation between the joint center and the femur and/or tibia or changes in the joint angle might occur. This has not been validated as we studied only native knees.

Clinically, a validated method to measure leg malalignment from standard knee radiographs would be very useful, as this would make a large proportion of full-limb radiographs unnecessary. A full-limb radiograph has several disadvantages, such as higher costs, more radiation, and the need for specialized equipment. However, it is important to question whether the mean observed error of 1.8° is of sufficient accuracy for clinical applications. Odenbring et al. (1993) suggested that a 3-degree accuracy in measuring the mechanical HKAA is sufficient, as this resembles the precision of a correction osteotomy. To our knowledge the scan–rescan error for determining the HKAA using full-limb radiographs is described in only one study including 8 cases. The authors reported a mean error of 1.3°, but their measurements are rounded to the full degree. Sanfridsson et al. (1996) found a correlation of 0.91 when comparing standard HKAA radiography with the novel QUESTOR method (using a specific positioning platform and software to perform and analyze the full-limb radiography). A statistically significant mean difference of 0.7° in HKAA between double and single leg weight-bearing full-limb radiographs was reported by Yazdanpanah et al. (2017) but they did not report the mean absolute error. More research is needed to investigate the scan–rescan reliability of the HKAA from full-limb radiographs.

Our study has a number of strengths. We used standardized clinical radiographs, with a protocol feasible in clinical care. We included an equal number of males and females. We tested a large number of FTA definitions using the same set of radiographs to directly compare their performance. Finally, we used a cross-fold validation experiment to test our predictions in unseen radiographs. The main limitation of our study is that the reliability of the gold standard (the HKAA) has been poorly studied. However, the HKAA is the most commonly used measurement to determine the mechanical angle of the lower extremity in both research studies and clinical care.

### Conclusion

We have developed an automated image analysis pipeline to calculate the FTA from standard knee radiographs. We directly compared multiple FTA definitions and tested their performance in predicting the HKAA, as measured from full-limb radiographs. The best-performing FTA definition correlated strongly with the HKAA and predicted it with high accuracy. The proposed image analysis pipeline can be used for epidemiological research on lower-limb alignment in cohorts with standard knee radiographs.  

All authors provided significant contribution to drafting and/or revising the manuscript and to research design. Additionally, all authors were involved in either acquisition, analysis, or interpretation of the data.

*Acta* thanks Kaj Knutson for help with peer review of this study.
